# Clinical Determinants and Prognostic Impact of Delayed Diagnosis in Wilson’s Disease

**DOI:** 10.3390/diagnostics15182358

**Published:** 2025-09-17

**Authors:** Agnieszka Antos, Maciej Niewada, Łukasz Kraiński, Anna Członkowska

**Affiliations:** 1 Second Department of Neurology, Institute of Psychiatry and Neurology, 02-957 Warsaw, Poland; 2 Department of Experimental and Clinical Pharmacology, Medical University of Warsaw, 02-097 Warsaw, Poland; 3 Decision Analysis and Support Unit, Warsaw School of Economics, 02-554 Warsaw, Poland

**Keywords:** Wilson’s disease, diagnostic delay, early diagnosis, prognosis, neurological deterioration

## Abstract

**Background**: Wilson’s disease (WD) is a rare hereditary disorder caused by pathological copper accumulation in multiple tissues. We aimed to assess the extent and determinants of diagnostic delay in WD adult patients treated at a Polish referral center and its impact on prognosis. **Methods**: We retrospectively analyzed 268 patients with WD diagnosed between 2008 and 2023. The duration of diagnostic delay was assessed in relation to sex, age, initial and diagnostic symptoms, liver enzyme levels, the aspartate aminotransferase-to-platelet ratio index, severity of hepatic and neurological manifestations, functional dependence, and family history of WD. Clinical outcomes included survival, liver transplantation, and neurological deterioration. **Results**: The mean diagnostic delay was 22.5 months (standard deviation (SD) 27.9). The shortest delay occurred in patients with hepatic presentation (18.3 months, SD 23.6), followed by neurological (26.8 months, SD 28.6), and psychiatric symptoms (65 months, SD 52.3). Longer delay correlated with older age at diagnosis and higher prevalence of neurological symptoms. In univariate analysis, diagnostic delay significantly increased the risk of neurological deterioration (*p* = 0.02). Patients with neurological or psychiatric symptoms, severe liver damage, and late-onset disease were at higher risk for adverse outcomes. **Conclusions**: Diagnostic delay was not associated with mortality or the composite endpoint but was linked to neurological deterioration. Surrogate markers suggested a possible relationship between delay and disease advancement. The absence of a clear association with prognosis may reflect challenges in adjusting for confounding factors such as treatment adherence.

## 1. Introduction

Wilson’s disease (WD) is a rare, autosomal recessive disorder resulting from mutations in the *ATP7B* gene, located on the long arm of chromosome 13 [1]. The *ATP7B* gene encodes a P-type ATPase (ATP7B) involved in intracellular copper transport. This enzyme is most highly expressed in the liver and participates in the excretion of copper into bile, its incorporation into ceruloplasmin (Cp), and the subsequent release of Cp-bound copper into systemic circulation [1,2,3,4,5,6]. Abnormalities in the structure of ATP7B result in excessive copper accumulation in the liver [1,2,3,4,5,6]. Hepatic copper overload leads to mitochondrial damage in hepatocytes, oxidative stress, and injury to cell membranes, lipids, and cellular DNA [5]. Consequently, this process triggers hepatocyte necrosis, chronic inflammation, and liver fibrosis at various stages of the disease, ultimately leading to clinical or subclinical manifestations of liver injury [5]. Further progression of WD is associated with the release of copper from the overloaded liver into systemic circulation in the form of free, non-ceruloplasmin-bound copper (NCC) [5]. This free copper gradually accumulates in other organs, including the brain, cornea, kidneys, heart, and bones. The resulting clinical manifestations include neuropsychiatric, ophthalmologic, cardiologic, renal, hematologic, and other systemic symptoms.

WD is a potentially treatable disorder with drugs leading to negative copper body balance (copper chelators or zinc salts leading to decreased copper absorption from the digestive tract) [1,2,3]. According to WD pathogenesis, the key factors for WD treatment outcome are early diagnosis and compliance with anti-copper treatment [1,2,3]. Diagnostic delay remains a critical issue in WD management [5,6,7]. Early recognition and treatment are crucial, as appropriate therapy can halt disease progression and often reverse symptoms, particularly those affecting hepatic and neurological functions [8]. However, pathological changes may become irreversible in advanced stages, highlighting the importance of timely and accurate diagnosis. In the majority of patients, clinical symptoms first manifest between the ages of 5 and 35 [5]. Hepatic symptoms most frequently appear first, ranging from asymptomatic liver enzyme elevation to cirrhosis or acute liver failure [3,5,9]. Neurological symptoms typically develop in the second or third decade of life, and include dysarthria, tremor, dystonia, parkinsonism, and ataxia [10,11,12,13,14]. Psychiatric disturbances—such as mood and personality disorders, cognitive decline, less frequently psychosis—are common and may precede other symptoms [15,16,17,18]. Characteristic ocular findings include the Kayser-Fleischer ring, especially in neurologic forms, and less often sunflower cataract [1,5,19]. Copper-related systemic effects may also involve kidneys, bones, skin, and the cardiovascular or endocrine systems [5,6]. Diagnosing WD can be challenging due to the lack of pathognomonic features and variability in presentation [1,3,5,8]. Core laboratory tests include serum ceruloplasmin, serum copper, and 24 h urinary copper excretion [1,3,6,20,21,22]. Neuroimaging, especially brain magnetic resonance imaging (MRI), often reveals characteristic lesions in the basal ganglia, thalamus, midbrain, and pons, even in preclinical stages [10,11,13,23]. Genetic testing for *ATP7B* gene mutations provides diagnostic confirmation [5,24,25]. Slit-lamp examination for the Kayser-Fleischer ring is diagnostically valuable, particularly in neurologic cases [1,5,19]. In rare, diagnostically unclear situations, radioactive copper incorporation tests may be used [26].

This study aims to evaluate the extent of diagnostic delay among WD patients treated at the Institute of Psychiatry and Neurology (IPiN) in Warsaw, who represent the majority of diagnosed WD cases in Poland [27]. We further aim to identify determinants of diagnostic delay and to assess its impact on disease progression and prognosis. Despite numerous international reports on this topic, data reflecting the Polish healthcare system and its diagnostic challenges are lacking. Delays in the diagnosis of WD remain a challenge and may negatively affect prognosis. Therefore, a better understanding of the factors contributing to diagnostic delay in WD and its clinical implications is essential for improving patient outcomes. Timely diagnosis and initiation of appropriate treatment are critical factors influencing outcomes in many genetic diseases. Delays in diagnosis can result in disease progression and irreversible complications.

## 2. Materials and Methods

### 2.1. Study Population and Data Collection

We retrospectively analyzed 268 adult patients diagnosed with WD between 2008 and 2023, who have remained under the continuous care of the Second Department of Neurology at IPiN. In 2005, an electronic database was established, based on the structure of the international EuroWilson registry, to systematically collect data on newly diagnosed cases of WD from multiple countries (www.eurowilson.org accessed on 7 April 2025). The IPiN registry comprises comprehensive data from patients managed at IPiN, including clinical, laboratory, radiological, genetic, and treatment-related information about WD and associated comorbidities. Data have been collected prospectively since 2005, following the established schedule of follow-up visits, with an assessment of neurological symptom severity and liver damage using standardized scales [27]. Data before 2005 were retrospectively integrated into the registry based on a thorough review of existing medical records.

**Figure 1:**
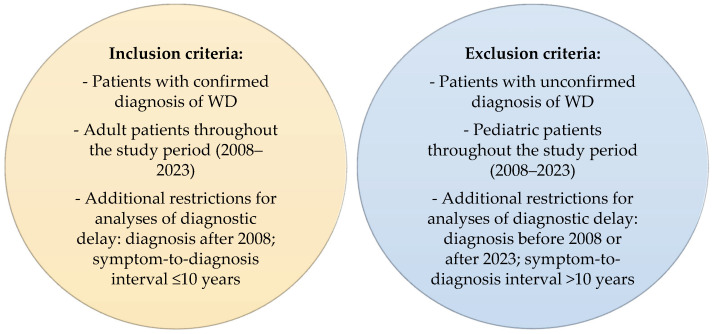
Inclusion and exclusion criteria for the study.

The inclusion criteria for the analyzed group were adulthood and confirmed WD diagnosis based on medical history, copper metabolism abnormalities, neuroimaging, ophthalmologic and genetic tests, and, in selected cases, the radioactive copper test. The study included adult patients, irrespective of their age at diagnosis. To ensure uniformity and comparability of the data, the analysis of diagnostic delay was restricted to patients diagnosed after 2008, as the routine application of the Unified Wilson’s Disease Rating Scale (UWDRS) for the assessment of neurological symptoms was implemented at IPiN that year. Additionally, the analysis was restricted to patients in whom the interval between patient-reported symptom onset and diagnosis did not exceed 10 years (120 months), to exclude cases with incidental or nonspecific symptoms unlikely to reflect the clinical course of WD and potentially confounding the assessment of diagnostic delay and symptom-related factors. Inclusion and exclusion criteria for the study are presented in Figure 1.

According to the pathogenesis of WD, we distinguished four main clinical phenotypes: hepatic—presenting only with liver symptoms; neurological—the presence of neurological symptoms, regardless of symptoms in other systems; psychiatric—presenting only with psychiatric symptoms; asymptomatic—the lack of clinical symptoms, with the diagnosis made based on family screening in patients with WD [20]. Patients were categorized according to the initial symptoms of WD as documented in their medical history, as well as the clinical features observed at the time of diagnosis. Initial symptoms of WD refer to the first manifestations of the disease noticed by the patient or recorded in medical documentation. Symptoms at diagnosis describe the patient’s condition at the time WD was confirmed, which often reflects disease progression. Both sets of data were obtained from medical records as well as patient recall. This distinction allows us to analyze diagnostic delay and its potential influence on disease severity at the time of diagnosis. Separate analyses were conducted for symptoms at disease onset and for those present at the time of diagnosis to distinguish early manifestations from the established clinical presentation at the time of diagnosis.

All adult patients meeting the inclusion criteria were included to illustrate the full spectrum of WD presentations in our cohort, including asymptomatic individuals identified through family screening. Consistently with published approaches, the definition of diagnostic delay was adopted as the time from the onset of the first clinical symptoms to the establishment of the diagnosis. This definition applies only to symptomatic patients; diagnostic delay in the asymptomatic group, where the diagnosis was based solely on copper metabolism and genetic testing due to positive family history, was not analyzed, since asymptomatic cases clearly lack an onset of clinical manifestations. Hepatic symptoms were categorized into five subgroups based on severity (scale 0–4), using clinical, laboratory, and imaging data: 0—no symptoms; 1—mild symptoms: elevated liver enzymes without abnormalities in abdominal ultrasound; 2—moderate symptoms: elevated liver enzymes + hepatomegaly/splenomegaly in abdominal ultrasound; 3—severe symptoms: elevated liver enzymes + changes in liver echostructure in abdominal ultrasound; 4—very severe symptoms: signs of portal hypertension, acute liver failure, or encephalopathy. Patients presenting with neurological symptoms were assessed using the UWDRS to evaluate the type and severity of neurological deficits and disability. The scale comprises three parts: Part I assesses the level of consciousness (0–3 points), Part II evaluates impairments in activities of daily living (0–39 points), and Part III measures neurological deficits on clinical examination (0–143 points) [14]. Additionally, these patients were classified into subtypes based on their clinical presentation: tremor-dominant, rigid, dystonic, mixed, with no predominant clinical form, and those with subtle symptoms who could not be assigned to any of the other categories.

Given the skewed distribution of diagnostic delay times, comparative analyses were conducted between patients in the first and fourth quartiles of the distribution, representing the shortest and longest intervals to diagnosis, respectively. We explored the following factors which may contribute to diagnostic delay in WD: gender, age and nature of the first symptoms, symptoms at diagnosis, concentrations of liver enzymes, aspartate aminotransferase to platelet ratio index (APRI) value, severity of hepatic symptoms, severity of neurological symptoms (UWDRS), patient functional dependence and family history of WD. The analysis was conducted in two steps. Initially, factors contributing to diagnostic delay were identified; subsequently, the impact of this delay on patient prognosis was evaluated. As part of the data collection, information regarding patients’ self-declared adherence to anti-copper therapy was also obtained.

To assess the impact of diagnostic delay on patient prognosis, the following outcomes were analyzed: composite endpoint (functional dependence, liver transplantation, or death), survival, and neurological deterioration (defined as deterioration of ≥1 point in UWDRS part II or ≥4 points in part III) [28].

### 2.2. Statistical Analysis

Quantitative variables were summarized using appropriate measures of central tendency (mean or median) and dispersion (standard deviation (SD) or interquartile range), depending on data distribution. Categorical variables were described using absolute counts and corresponding percentages.

Student’s *t*-test for independent or paired samples was used to test statistical hypotheses for continuous variables. In cases where the assumptions of normal distribution were not met, non-parametric tests such as the Wilcoxon signed-rank test or the Kruskal–Wallis test were applied. For qualitative variables, the chi-square test was used to analyze differences between groups. To assess the effect of a single independent variable with multiple levels on a dependent variable, a one-way analysis of variance (ANOVA) was employed. Statistical significance was considered for *p*-values equal to or less than 0.05.

The multivariate analysis was based on logistic regression models for discrete variables and Cox regression models for time-to-event variables, following prior verification of the proportional hazards assumption. Explanatory variables for the multivariate analysis were selected based on clinically established prognostic relevance, not only univariate results. They included sex, age at symptom onset, symptom type at diagnosis, functional dependence at diagnosis, treatment regularity, and diagnostic delay.

## 3. Results

### 3.1. Patient Characteristics

The study group included 268 patients diagnosed with WD, comprising 138 women and 130 men. The mean diagnostic delay for the entire group was 22.5 months. The mean age of the participants was 39 years, and the mean age at diagnosis was 29 years. The age at diagnosis varied depending on the clinical presentation at onset. Self-reported adherence to treatment protocols was high, with 97% of patients (261/268) declaring compliance. Results of patients’ characteristics are summarized in Table 1.

### 3.2. Risk Factors for Diagnostic Delay

From the analyzed factors that may contribute to diagnostic delay in WD, the type of dominant symptoms at diagnosis was significantly associated with the length of the diagnostic delay (Table 1). Patients with hepatic symptoms had the shortest delay, on average 18.3 months (*p* = 0.01), followed by those with neurological symptoms (26.8 months; *p* = 0.03), while the longest delay was observed in patients with psychiatric symptoms, 65 months on average (*p* = 0.03). Comparing patients with the shortest and longest time to diagnosis, longer diagnostic delay was associated with older age at diagnosis (32.2 vs. 26.9 years; *p* = 0.01), higher rate of neurological manifestations both at disease onset (57.1% vs. 30.6%, *p* = 0.001) and at the time of diagnosis (54.3% vs. 25%, *p* < 0.001) (Table 2).

### 3.3. Impact of Diagnostic Delay on Clinical Outcomes

In univariate analysis, there was no significant association between diagnostic delay (comparison of the 1st and 4th quartile) and the occurrence of the composite endpoint (*p* = 0.75), which was confirmed in multivariate models. In logistic regression analysis, diagnostic delay was not a significant predictor of the composite endpoint (*p* = 0.44). However, significant predictors included older age at symptom onset (*p* = 0.002, OR = 1.05), hepatic symptoms at diagnosis (*p* = 0.01, OR = 3.13), and functional dependence at the time of diagnosis (*p* < 0.001, OR = 7.69). In Cox proportional hazards regression, diagnostic delay also did not significantly influence the time to the composite endpoint (*p* = 0.63). Factors associated with a significantly increased hazard of reaching the endpoint were the presence of hepatic symptoms (*p* = 0.03, HR = 1.97), psychiatric symptoms (*p* = 0.02, HR = 2.61), and functional dependence at diagnosis (*p* < 0.001, HR = 6.01).

In the survival analysis, patients who died had a slightly longer diagnostic delay (26.8 vs. 22.2 months), although this difference was not statistically significant (*p* = 0.49). Diagnostic delay was not significantly associated with the risk of death in univariate analyses (*p* = 0.32), logistic regression (*p* = 0.49), or Cox regression (*p* = 0.54). In logistic regression, significant predictors of death were older age at onset of symptoms (*p* = 0.002, OR = 1.08) and functional dependence at diagnosis (*p* < 0.001, OR = 12.5). In Cox regression, the risk of death increased by 6% with each additional year of age at symptom onset (*p* = 0.01, HR = 1.06), and functional dependence at the time of diagnosis remained a strong negative prognostic factor (*p* < 0.001, HR = 7.69).

During the follow-up period, 15% of patients (41 out of 268) experienced neurological deterioration as assessed using the UWDRS. Although these patients had a longer diagnostic delay on average (25.7 vs. 21.9 months), the difference was not statistically significant (*p* = 0.43). In univariate analysis, diagnostic delay was significantly associated with increased risk of neurological deterioration (*p* = 0.02), but this was not confirmed in multivariate models. In logistic regression, diagnostic delay did not significantly predict neurological deterioration (*p* = 0.49). However, female sex (*p* = 0.03, OR = 2.5) and the presence of neurological symptoms at diagnosis (*p* < 0.001, OR = 38) were both associated with a higher likelihood of deterioration. In Cox regression, diagnostic delay did not significantly affect the time to neurological deterioration (*p* = 0.78). Significant predictors of faster deterioration included female sex (*p* = 0.03, HR = 2.08) and neurological symptoms at diagnosis (*p* < 0.001, HR = 9.2).

To sum up: in univariate analysis, diagnostic delay was linked only to neurological deterioration, but not to the composite endpoint or mortality (Table 3); in multivariate logistic regression, diagnostic delay was not a predictor of adverse outcomes, whereas older age, hepatic and neurological symptoms, and functional dependence at diagnosis consistently indicated poorer prognosis (Table 4); in Cox regression, poorer outcome was predicted by older age, female sex, hepatic, neurological and psychiatric symptoms, as well as functional dependence deterioration, but not by diagnostic delay (Table 5).

## 4. Discussion

Confirming the WD diagnosis remains a challenge, with the average time between the onset of symptoms and diagnosis usually exceeding 2 years [29,30,31,32]. Delayed diagnosis and, consequently, delayed treatment initiation are significant issues that influence the prognosis of patients with WD [31,32]. Several factors contribute to this delay, including: the nature of the disease—nonspecific symptoms of WD can be confused with more common conditions; social factors—patients may avoid medical consultations due to fear of negative consequences and stigmatization; lack of physician experience—diagnosing rare diseases such as WD is more difficult, as healthcare providers have less experience with these conditions; and the healthcare system—inequalities in access to specialists and diagnostic tests prolong the time required to establish a diagnosis.

As WD is primarily a hepatic disorder, liver damage is highly characteristic of the disease. Liver dysfunction is considered to occur in all patients and represents one of the early stages in the disease’s progression, followed by damage to other organs, including the brain [5]. The type of symptoms at the time of diagnosis significantly influenced the time to diagnosis. The shortest time to diagnosis was observed in patients with hepatic symptoms, followed by those with neurological symptoms, while the longest delay was noted in patients with psychiatric symptoms. These results are consistent with the literature—patients presenting with hepatic symptoms at diagnosis experience shorter diagnostic delays than those with neuropsychiatric symptoms [5,29]. Hepatic manifestations of WD typically emerge earlier in life than neurological or psychiatric symptoms, which usually develop later as a result of central nervous system involvement. However, the temporal relationship between symptom onset and diagnostic delay is often difficult to delineate. Early hepatic signs may be subtle, nonspecific, or clinically underestimated, leading to postponed recognition until more overt neurological or psychiatric features arise. Additionally, the severity and dynamic nature of WD—characterized by fluctuating symptom expression and multisystem involvement—further complicate efforts to distinguish between true disease progression and delays in clinical detection.

In both univariate and multivariate analyses, it was found that diagnostic delay was not associated with the composite endpoint or mortality alone. The results are surprising. Hu et al. demonstrated better outcomes in patients with shorter diagnostic delays in their study on WD [33]. In the study from 2005, which primarily focused on neurological cases, higher mortality was found among WD patients compared to the general population, with most deaths attributed to late diagnosis, lack of appropriate pharmacological treatment, and the use of adjunctive medications [34]. In our study, adherence to treatment regimens may have contributed to these ambiguous findings, as almost all patients (261/268) reported following treatment protocols, which could not be objectively verified [35].

Although diagnostic delay was significantly associated with neurological deterioration in univariate analysis, this relationship did not persist after adjustment for other variables in multivariate models. The literature on the impact of diagnostic delay on the risk of neurological deterioration is inconsistent. Ziemssen et al. emphasized the significant effect of diagnostic delay on neurological decline [36]. Litwin et al. found that severe baseline neurological deficits significantly contributed to further deterioration, while factors such as disease duration and liver damage did not differ between the groups with and without neurological worsening [37]. Stanković et al. concluded that diagnostic delay does not increase the risk of neurological deterioration in patients who consistently adhere to treatment [38]. Similarly, Prashanth et al. found no significant association between diagnostic delay and worsening neurological status [39]. Potential factors influencing the divergent results in the literature include the limitations of the study methodology, such as the challenges in precisely identifying the first symptoms of WD, as well as the retrospective nature of the analysis. The difficulty in determining the exact significance of diagnostic delay arises from the nonspecific nature of WD symptoms and the absence of pathognomonic signs. In publications, experts face similar challenges in determining diagnostic delay, primarily due to inconsistencies in identifying the first signs of the disease. The symptoms of WD are nonspecific and can easily be mistaken for other conditions. For instance, abdominal pain, neurological symptoms such as tremors, or psychiatric symptoms such as depression or personality changes do not unequivocally point to WD. No two patients exhibit the same phenotype, even among siblings with WD (including twins). The disease’s variable progression, different stages of the disease at the time of diagnosis, variable adherence to therapeutic recommendations, and other prognostic factors (genetic or environmental) can complicate the determination of the impact of diagnostic delay on the prognosis. Therefore, it should not be concluded that diagnostic delay does not influence the prognosis of patients with WD. In our study, based on the available data, we were unable to confirm an association between diagnostic delay and prognosis.

Worse outcomes in patients with neurological or psychiatric symptoms, more severe liver damage, and in those who were non-independent at diagnosis indirectly suggest a negative impact of diagnostic delay on the prognosis of WD patients. These symptoms are indicative of advanced disease and are a result of prolonged disease progression without appropriate treatment. Reducing the time to diagnosis may improve the prognosis for patients and reduce the risk of complications associated with advanced stages of the disease.

Primary care physicians should maintain a high index of suspicion in patients with unexplained liver abnormalities, neuropsychiatric symptoms, or a combination of otherwise nonspecific manifestations. Psychiatric features—including mood disorders, behavioral changes, or cognitive decline—may precede more classical hepatic or neurological signs, particularly in younger individuals. Initial evaluation in the primary care setting may involve liver function tests. Abnormal results should trigger referral to a specialized center for comprehensive evaluation. Such abnormalities should also raise consideration of WD in the differential diagnosis, particularly when neurological or psychiatric symptoms in young adults accompany liver test abnormalities. In a specialized center, a comprehensive assessment using neuroimaging, ophthalmologic examination, and genetic testing can confirm the diagnosis and guide early treatment. At our center, the diagnostic work-up for WD follows a stepwise approach in line with current recommendations [27,40,41,42]. The Leipzig score is used as a supportive tool in the diagnostic process, integrating clinical symptoms, biochemical parameters, neuroimaging, ophthalmologic findings, and genetic testing [21]. Initial laboratory evaluation includes liver function tests and assessment of copper metabolism, including serum ceruloplasmin, total serum copper, and 24 h urinary copper excretion. Brain MRI is performed to detect structural abnormalities, which are highly prevalent in neurological forms and can also be present in asymptomatic cases. Ophthalmologic examination includes slit-lamp evaluation for the presence of Kayser-Fleischer rings. Genetic testing confirms the presence of biallelic pathogenic variants in the *ATP7B* gene, although a subset of patients elects to undergo this testing independently. In selected cases with diagnostic uncertainty, the radioactive copper incorporation test can be applied to assess the liver’s ability to incorporate copper into apoceruloplasmin. Once WD is diagnosed, treatment is promptly initiated, and patients continue to receive long-term follow-up and management within a specialized neurological outpatient clinic.

This study has several limitations. The rarity of WD limits the sample size (*n* = 268), reducing statistical power and hindering the detection of significant associations or effective adjustment for confounding variables. Both diagnostic delay and prognosis in WD are multifactorial and influenced by unmeasured individual factors (e.g., health literacy, social support, comorbidities) and systemic factors (e.g., diagnostic availability, physician awareness), which were not included in the analysis. It is a retrospective study; initial symptoms were assessed retrospectively based on patient recall, which may be biased, particularly given the nonspecific and variable nature of early WD manifestations, or on medical records that were sometimes dated and potentially incomplete. It should be noted that the cohort did not include patients with the most severe hepatic manifestations, which may limit the applicability of our findings to this subgroup. However, IPiN is the largest adult referral center for patients with WD in Poland, which supports the representativeness of the studied population. To ensure greater data homogeneity, the analysis was limited to patients under the care of the II Department of Neurology IPiN, who were diagnosed with WD between 2008 and 2023, with a diagnostic delay of ≤120 months. Including cases with longer delays could have led to misclassification of incidental or unrelated symptoms as disease-related. In WD, defining a single standardized measure of diagnostic delay with clear prognostic relevance remains challenging. As the disease progresses, comorbidities and secondary factors, such as treatment non-adherence, further complicate the clinical picture, underscoring the need for individualized interpretation in both diagnosis and prognosis.

## 5. Conclusions

Key findings from this study indicate that the diagnostic process in WD is often prolonged, with a median delay of 22.5 months. This delay is largely dependent on the type and timing of symptom manifestation—both those initially reported and those present at the time of diagnosis. Older patients, who tended to develop symptoms later and with less severity, experienced longer diagnostic delays. Diagnostic delay was significantly associated with neurological deterioration in univariate analysis, but this relationship did not persist in multivariate models. Although the study did not demonstrate a direct association between diagnostic delay and patient prognosis, surrogate indicators of disease advancement suggested a potential link. This lack of a clear relationship may reflect the difficulty of adjusting for multiple confounding factors, particularly treatment adherence and proper long-term medication use. The long-lasting and variable course of WD, combined with the lack of symptoms that are unique to the disease, makes it challenging to establish the exact duration of diagnostic delay and to evaluate its precise impact on patient prognosis.

## Figures and Tables

**Table 1:** Characteristics of the study group and diagnostic delay.

Category	Subgroup/Variable	Results
**Baseline characteristics**		
Sex distribution	Female (*n*)	138
	Male (*n*)	130
Mean age at diagnosis	Hepatic	28.8 years old (SD 11.0)
	Neurological	33.7 years old (SD 11.0)
	Psychiatric	32.8 years old (SD 8.8)
	Asymptomatic	26.1 years old (SD 13.2)
Initial symptoms	Hepatic	60 (22.4%)
	Neurological	196 (73.1%)
	Psychiatric	10 (3.7%)
	None	2 (0.7%)
Clinical form at diagnosis	Hepatic	103 (38.4%)
	Neurological	120 (44.8%)
	Psychiatric	2 (0.7%)
	Asymptomatic	43 (16.0%)
Family history	Positive	61 (22.8%)
	Negative	207 (77.2%)
Declared treatment adherence	Adherent	261 (97.4%)
	Non-adherent	7 (2.6%)
**Diagnostic delay**		
Mean diagnostic delay	entire group	22.5 months (SD 27.9)
Diagnostic delay	0–12 months	149 (55.6%)
	13–24 months	40 (14.9%)
	25–36 months	19 (7.1%)
	37–48 months	18 (6.7%)
	49–60 months	8 (3.0%)
	61–72 months	9 (3.4%)
	73–84 months	10 (3.7%)
	85–96 months	3 (1.1%)
	97–108 months	7 (2.6%)
	109–120 months	5 (1.9%)
Mean diagnostic delay ^	Female	21.7 months (SD 27.7)
	Male	23.3 months (SD 28.2)
Symptoms at diagnosis and delay *	Hepatic	18.3 months (SD 23.6)
	Neurological	26.8 months (SD 28.6)
	Psychiatric	65 months (SD 52.3)

^—*p*-value = 0.65; *—*p*-value < 0.05; SD—standard deviation.

**Table 2:** Statistically significant differences between patients with early (Q1) and late (Q4) diagnosis of WD.

Factor	Patients in Q1 (*n*)	Q1	Patients in Q4 (*n*)	Q4	*p*-Value
Mean age at WD onset (years)	72	26.9	70	32.2	0.01
Neurological symptoms as first manifestation (%)	72	30.60%	70	57.10%	<0.01
Neurological symptoms at diagnosis (%)	72	25.00%	70	54.30%	<0.01

Q1—first diagnostic delay quartile (earliest diagnoses); Q4—fourth quartile (latest diagnoses).

**Table 3:** Impact of diagnostic delay on patient outcomes—univariate analyses.

Delay in Diagnosis (Quartiles)	Outcome	Yes	No	Total Patients Number	*p*-Value
Q1	Composite endpoint	14	58	72	
Q4	Composite endpoint	13	47	60	0.75
Q1	Death	4	68	72	
Q4	Death	7	63	70	0.32
Q1	Neurological worsening	5	67	72	
Q4	Neurological worsening	14	56	70	0.02

Composite endpoint—Death/Liver transplantation/Dependency; Q1—first diagnostic delay quartile (earliest diagnoses); Q4—fourth quartile (latest diagnoses).

**Table 4:** Logistic regression of prognostic factors in WD, including diagnostic delay.

Variable	Composite Endpoint—OR (95% CI)	*p*-Value	Death—OR (95% CI)	*p*-Value	Neurological Deterioration—OR (95% CI)	*p*-Value
Female sex	0.73 (0.36–1.46)	0.37	0.94 (0.30–2.91)	0.91	2.50 (1.14–5.88)	0.03
Hepatic symptoms at diagnosis	3.13 (1.40–7.64)	0.01	1.83 (0.58–6.59)	0.32	1.43 (0.64–3.31)	0.98
Neurological symptoms at diagnosis	1.07 (0.48–2.36)	0.87	0.85 (0.21–3.43)	0.82	38.00 (10.30–248.45)	<0.001
Psychiatric symptoms at diagnosis	0.95 (0.21–3.53)	0.94	1.97 (0.31–10.23)	0.44	2.08 (0.72–5.93)	0.17
Age at symptom onset (years)	1.05 (1.02–1.09)	0.002	1.08 (1.03–1.13)	0.002	0.99 (0.95–1.03)	0.59
Dependency at diagnosis	7.69 (2.86–20.00)	<0.001	12.50 (3.57–50.00)	<0.001	1.36 (0.49–4.18)	0.57
Treatment compliance	N/A	0.99	N/A	0.99	N/A	0.99
Diagnostic delay (months)	0.99 (0.98–1.01)	0.44	1.01 (0.99–1.03)	0.49	1.00 (0.98–1.01)	0.49

Composite endpoint—Death/Liver transplantation/Dependency; OR—odds ratio; CI—confidence interval; N/A—not applicable.

**Table 5:** Cox regression of prognostic factors in WD, including diagnostic delay.

Variable	Composite Endpoint—HR (95% CI)	*p*-Value	Death—HR (95% CI)	*p*-Value	Neurological Deterioration—HR (95% CI)	*p*-Value
Female sex	0.66 (0.38–1.16)	0.15	0.84 (0.31–2.32)	0.74	2.08 (1.05–4.00)	0.03
Hepatic symptoms at diagnosis	1.97 (1.08–3.61)	0.03	1.51 (0.54–4.19)	0.43	1.58 (0.80–3.10)	0.19
Neurological symptoms at diagnosis	1.73 (0.86–3.49)	0.13	0.93 (0.23–3.69)	0.92	9.20 (3.86–21.92)	<0.001
Psychiatric symptoms at diagnosis	2.61 (1.20–5.66)	0.02	1.36 (0.34–5.46)	0.67	1.87 (0.85–4.10)	0.12
Age at symptom onset (years)	1.01 (0.99–1.04)	0.29	1.06 (1.02–1.11)	0.01	1.00 (0.97–1.03)	0.94
Dependency at diagnosis	6.01 (3.23–11.11)	<0.001	7.69 (2.38–25.00)	<0.001	1.64 (0.66–4.03)	0.29
Treatment compliance	N/A	0.99	N/A	0.99	N/A	0.99
Diagnostic delay (months)	1.00 (0.99–1.01)	0.63	1.01 (0.99–1.02)	0.54	1.00 (0.99–1.01)	0.78

Composite endpoint—Death/Liver transplant/Dependency; HR—hazard ratio; CI—confidence interval; N/A—not applicable.

## Data Availability

The data presented in this study are available on reasonable request from the corresponding author. The data are not publicly available due to privacy and ethical restrictions, as they are stored in the institutional clinical database of the Second Department of Neurology, Institute of Psychiatry and Neurology, Warsaw, Poland.
